# Extracellular Vesicular Delta‐Like Ligand 3 and Subtype Transcription Factors for Small Cell Lung Cancer Diagnosis

**DOI:** 10.1002/advs.202416711

**Published:** 2025-04-26

**Authors:** Hong Li, Chi‐Ling Chiang, Kwang Joo Kwak, Hsin‐Lun Lee, Xinyu Wang, Giulia Romano, Michela Saviana, Robin Toft, Tai‐Shan Cheng, Yuehshih Chang, Bi‐Da Hsiang, Guan‐Wan Liu, Xiaokui Mo, Yifan Ma, Junjie Pan, Xilal Y. Rima, Truc Nguyen Kim, Eduardo Reategui, Chia‐Ning Shen, Yeh‐Shiu Chu, Carlo Croce, Peter Mu‐Hsin Chang, Yi‐Chen Yeh, David P. Carbone, Chi‐Ying F. Huang, Chi‐Lu Chiang, Patrick Nana‐Sinkam, L. James Lee

**Affiliations:** ^1^ Department of Chemical and Biomolecular Engineering The Ohio State University Columbus OH 43210 USA; ^2^ Institute of Biopharmaceutical Sciences National Yang Ming Chiao Tung University Taipei 11221 Taiwan; ^3^ Spot Biosystems Ltd. Columbus OH 43212 USA; ^4^ Department of Radiology School of Medicine College of Medicine Taipei Medical University Taipei 11031 Taiwan; ^5^ Department of Radiation Oncology Taipei Medical University Hospital Taipei 11031 Taiwan; ^6^ Genomic Research Center Academia Sinica Taipei 11529 Taiwan; ^7^ The Ph.D. Program for Translational Medicine College of Medical Science and Technology Taipei Medical University and Academia Sinica Taipei 11031 Taiwan; ^8^ Division of Pulmonary Diseases and Critical Care Medicine Virginia Commonwealth University Richmond VA 23284 USA; ^9^ Division of Hematology and Oncology Department of Internal Medicine Keelung Chang Gung Memorial Hospital Keelung 20401 Taiwan; ^10^ School of Medicine College of Traditional Chinese Medicine Chang Gung University Taoyuan 33302 Taiwan; ^11^ Center for Biostatistics The Ohio State University Columbus OH 43210 USA; ^12^ Department of Biomedical Engineering The Ohio State University Columbus OH 43210 USA; ^13^ Brain Research Center National Yang Ming Chiao Tung University Taipei 11221 Taiwan; ^14^ College of Medicine The Ohio State University Columbus OH 43210 USA; ^15^ School of Medicine National Yang Ming Chiao Tung University Taipei 11221 Taiwan; ^16^ Department of Oncology Taipei Veterans General Hospital Taipei 11217 Taiwan; ^17^ Department of Pathology and Laboratory Medicine Taipei Veterans General Hospital Taipei 11217 Taiwan; ^18^ Department of Chest Medicine Taipei Veterans General Hospital Taipei 11217 Taiwan

**Keywords:** DLL3 mRNA in exosomes and protein in tumor‐associated extracellular vesicles, immune lipoplex nanoparticle biochip assay, POU2F3 and ASCL1 mRNA in exosomes, SCLC detection, single extracellular vesicle analysis

## Abstract

Small cell lung cancer (SCLC) is associated with high mortality and limited therapeutic options. There is increasing recognition that SCLC harbors molecular heterogeneity. Using a new liquid biopsy assay, it is demonstrated that SCLC subtypes, as determined by patient tumor tissue staining and cell lines, can be accurately identified by measuring the mRNA expression of subtype transcription factors (ASCL1, POU2F3, and NEUROD1) in circulating exosome‐rich extracellular vesicles (Exo). Additionally, upregulation of *Delta‐like ligand 3* (DLL3) mRNA in Exo and its membrane protein (mProtein) in extracellular vesicles associated with tumor (tEV) may distinguish both limited‐ and extensive‐stage SCLC patients from high‐risk smokers, with AUC/ROC values of 0.836 and 0.839, respectively. By incorporating Exo‐ASCL1 and Exo‐POU2F3 mRNA expression with DLL3 Exo‐mRNA/tEV‐mProtein expression, the classifier enhances the AUC/ROC to 0.912 and 0.963 for limited‐ and extensive‐stage SCLC patients, respectively.

## Graphical abstract

Small cell lung cancer (SCLC) harbors high molecular heterogeneity. Using a new liquid biopsy assay, SCLC subtypes can be identified by measuring mRNA of subtype transcription factors (ASCL1, POU2F3, and NEUROD1) in exosome‐rich extracellular vesicles. Combining with *Delta‐like ligand 3* (DLL3) mRNA in exosomes and its membrane protein in extracellular vesicle‐associated with tumor may distinguish SCLC patients from high‐risk smokers.

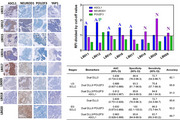

## 1 Introduction

Small cell lung cancer (SCLC) is a highly aggressive and poorly differentiated neuroendocrine (NE) cancer, accounting for 13% of lung cancer cases and carrying a high mortality (< 7% 5‐year survival).^[^ ^1^, ^2^, ^3^ ^]^ The high mortality is partially attributable to the lack of early detection methods,^[^ ^4^, ^5^ ^]^ lack of symptoms in early‐stage SCLC patients and limited therapeutic options. Additionally, simple imaging methods such as computed tomography (CT) scans are often insensitive, leading to diagnosis at later stages. While tissue biopsy and advanced imaging methods, such as magnetic resonance imaging (MRI) and positron emission tomography (PET) scans, offer greater diagnostic accuracy, they are costly and invasive, making them impractical for large‐scale screening.

Chemoradiotherapy is initially effective in treating SCLC; however, recurrence typically occurs within 6 months.^[^ ^6^ ^]^ Despite the emergence of newer therapeutic strategies, their efficacy remains limited, highlighting the need for a deeper understanding of SCLC's molecular heterogeneity and plasticity.

Recently, four distinct molecular subtypes of SCLC have been identified based on the expression of key transcription factors: *achaete‐scute homolog 1* (ASCL1), *neurogenic differentiation factor 1* (NEUROD1), *POU class 2 homeobox 3* (POU2F3) and *yes‐associated protein 1* (YAP1), classified as SCLC‐A, ‐N, ‐P, and ‐Y, respectively.^[^ ^7^ ^]^ Additionally, an SCLC‐I subtype has been characterized, representing SCLC populations with low ASCL1, NEUROD1, and POU2F3 expression, accompanied by an inflammatory gene signature.^[^ ^8^ ^]^ Emerging data suggest that these subtypes may exhibit different responses to therapy.^[^ ^8^, ^9^, ^10^ ^]^

Deregulation of the NOTCH signal pathway is a critical event in SCLC tumorigenesis, disease progression, and chemoresistance.^[^ ^3^, ^11^, ^12^ ^]^ NOTCH inactivating mutations occur in ≈25–28% of SCLC cases, leading to loss of the NOTCH signaling function.^[^ ^3^, ^13^ ^]^ Notably, DLL3, an inhibitory NOTCH ligand, is expressed in more than 75% of SCLC, with most patients exhibiting high DLL3 expression.^[^ ^14^ ^]^ As a result, DLL3 has been identified as a potential therapeutic target in high‐grade neuroendocrine (NE) carcinomas, including SCLC and large‐cell neuroendocrine carcinoma, based on patient‐derived xenograft models.^[^ ^15^ ^]^

A DLL3‐targeted antibody‐drug conjugate, rovalpituzumab tesirine (Rova‐T), has demonstrated antitumor efficacy in vivo and showed encouraging clinical outcomes in first‐in‐human phase I trials for relapsed SCLC.^[^ ^16^, ^17^ ^]^ However, the phase III trial for assessing Rova‐T as a first‐line maintenance therapy in advanced SCLC was unsuccessful due to severe side effects.^[^ ^18^ ^]^ Despite this setback, DLL3 remains a promising target for SCLC. Currently, DLL3‐targeted immune checkpoint inhibitors,^[^ ^17^ ^]^ T cell engagers such as Tarlatamab from Amgen,^[^ ^19^, ^20^ ^]^ and CAR‐T‐based drugs^[^ ^21^ ^]^ are in clinical trials, showing promising results.

Our understanding of the molecular mechanisms underlying SCLC is primarily derived from preclinical models and analyses of tumor tissues and cancer cells obtained through tissue biopsy and surgical resection. Unlike tissue biopsy, blood‐based liquid biopsy offers a minimally invasive and low‐cost alternative for cancer detection and surveillance. Circulating tumor cells,^[^ ^22^ ^]^ blood proteins,^[^ ^23^ ^]^ and blood microRNAs^[^ ^24^, ^25^ ^]^ have all been investigated in both non‐small cell lung cancer and SCLC. However, none have yet demonstrated sufficient potential to inform clinical care in SCLC.

Extracellular RNAs and proteins are stable in blood and other body fluids, partly due to their encapsulation within cell‐secreted extracellular vesicles (EVs) such as exosomes and microvesicles.^[^ ^26^, ^27^, ^28^, ^29^ ^]^ Capturing EVs and analyzing their encapsulated contents is a promising strategy for non‐invasive cancer detection.^[^ ^30^, ^31^, ^32^ ^]^ Most EV markers studied today are non‐coding microRNAs and proteins identified through patient blood profiling.^[^ ^33^, ^34^, ^35^ ^]^ While often highly expressed, individual microRNAs and proteins are often not directly linked to tumorigenesis, necessitating the use of large panels to establish statistically meaningful classifiers.^[^ ^36^, ^37^ ^]^ However, classifiers identified in one patient cohort may not always be reproducible in others.

In contrast, extracellular mRNAs in EVs, particularly those associated with oncogenes or their transcription factors, have shown strong potential as diagnostic or prognostic cancer biomarkers. For lung cancer, examples include extracellular *Thyroid transcription factor‐1* (TTF1) and *Transketolase‐like‐1* (TKTL1) mRNAs for diagnosing non‐small cell lung adenocarcinoma,^[^ ^38^ ^]^ as well as extracellular *programmed cell death protein‐1* (PD‐1) and *Programmed death ligand‐1* (PD‐L1) mRNAs for diagnosing non‐small cell lung cancer and predicting response to PD‐1/PD‐L1 immunotherapy.^[^ ^39^ ^]^

Here, we present an **I**mmune **L**ipoplex **N**anoparticle (**ILN**) biochip platform capable of detecting both mRNA and membrane protein (mProtein) at the single EV level in selected EV subpopulations captured via immune‐affinity sorting from human serum/plasma samples. Using the ILN biochip assay, we measured DLL3 mRNA expression with molecular beacon (MB) probes in CD63/CD9/CD81+ exosome‐rich EVs (Exo), captured using a mixture of CD63, CD9 and CD81 monoclonal antibodies. Corresponding mProtein expression was assessed using fluorescence‐labeled DLL3 antibody in DLL3/epithelial cell adhesion molecule (EpCAM)/receptor tyrosine kinase‐like orphan receptor 1 (ROR1)^+^ tumor‐associated EVs (tEV), captured with a mixture of DLL3, EpCAM, and ROR1 monoclonal antibodies.

This assay demonstrates significant differences between SCLC patients, healthy donors, and high‐risk smokers (HRS) using clinical patient samples from medical centers in multiple hospitals. Additionally, it successfully identified SCLC molecular subtypes. A combination of EV DLL3 and subtype transcription factor expressions provides excellent classification performance (AUC/ROC of 0.9∼0.95) when distinguishing SCLC patients from HRS.

SCLC represents an aggressive subtype of lung cancer with limited therapeutic options and yet‐to‐be‐identified strategies for early detection. The recent observation that SCLC may harbor molecular heterogeneity creates an opportunity for better elucidating drivers of disease progression that may be leveraged for therapeutic development. Here, we present a novel ILN biochip technology that can simultaneously identify biomarkers in serum/plasma samples offering a unique liquid biopsy tool for both complementary and companion diagnosis of SCLC. Further, it also creates new opportunities to investigate potential biological roles for extracellular vesicles as surrogate markers of molecular subtypes.

## 2 Results

### 2.1 Design and Characterization of ILN Biochip Assay

**Figure 1:**
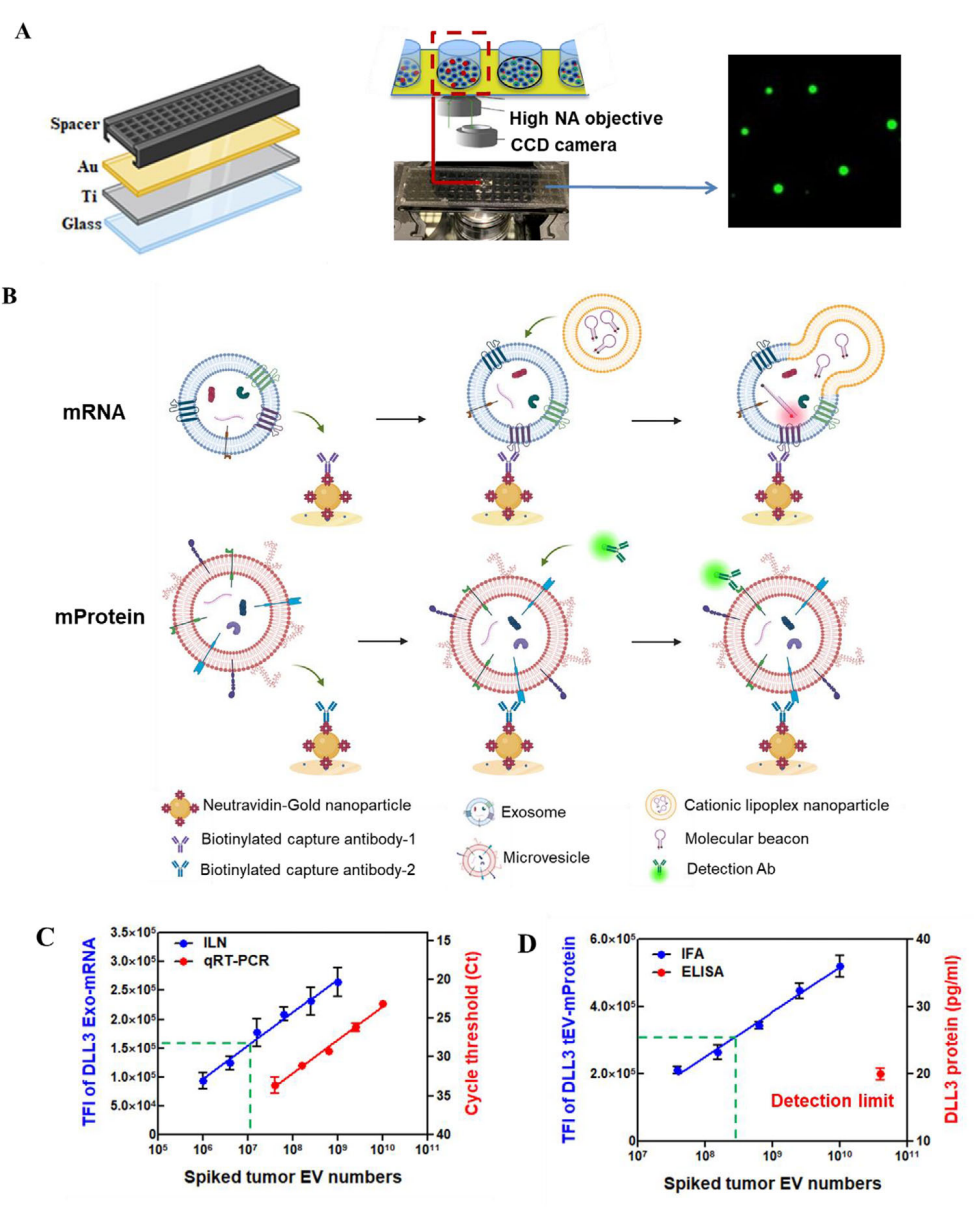
Immune Lipid Nanoparticle (ILN) biochip assay. A) Biochip assembly and images for Total Internal Fluorescence Reflection (TIFR) detection B) Schematic of the ILN biochip assay for EV sorting to detect Exo‐mRNA and tEV‐mProtein. The measured TFI is based on 100 TIRF images, with each image covers a surface area of 80 × 80 µm. C,D) Calibration curves for DLL3 mRNA expression in exosom‐rich extracellular vesicles (Exo) and DLL3 mProtein expression in tumor associated extracellular vesicular (tEV) were established using H82‐derived EVs spiked into 1 mL healthy donor serum (*n* = 3). Cut‐off values to distinguish SCLC from high‐risk smokers (HRS) are marked. TFI; Total Fluorescence Intensity. *p‐*values were determined by the two‐way ANOVA test. *p < 0.05 and **p < 0.01.

The design and schematic of the ILN biochip assays are shown in **Figure** **1**A,B. A thin titanium/gold (Ti/Au) coated cover glass was treated with a linker solution^[^ ^40^ ^]^ composed of 1‐thiahexa (ethylene oxide) lipid, WC14, biotin‐PEG‐SH and a lateral spacer, 𝛽‐mercaptoethanol with a molar ratio of 5:2:93. This surface was then tethered with 40 nm sized neutravidin gold nanoparticles via biotin‐streptavidin interactions. EV subpopulations were captured using biotin‐conjugated capture antibodies (such as CD63/CD81/CD9 or DLL3/EpCAM/ROR1). DLL3 mRNA expression was determined using a specifically designed molecular beacon encapsulated in cationic lipid nanoparticles (CLN‐MB), while DLL3 mProtein expression was determined using a fluorescence‐conjugated primary antibody. High‐resolution total internal reflection fluorescence (TIRF) microscopy was used to capture 100 fluorescence images per well, and the total fluorescence intensity (TFI) was quantified through image analysis.

For high‐throughput screening applications, EVs were isolated using a commercially available kit (Invitrogen plasma total exosome isolation kit, TEI kit) with slight modifications. EVs from SCLC patients had an average particle size of 50–200 nm, with EV numbers ranging from 1.5E10 to 8E10 particles per mL. EV concentrations did not differ between healthy donors and SCLC patient plasma/serum (Figure S1, Supporting Information). We saturated the biochip surface with a high number of EVs (i.e., the EV count in the sample was 10–100‐fold greater than the capture capacity of the surface), ensuring that variations in vesicle particle concentration among samples did not affect the measurement. Figure S2 (Supporting Information) shows saturated EVs covering the entire ILN biochip surface.

Nanoparticle tracking analysis (NTA) was used to measure the concentration of EVs derived from H82 SCLC cells, which were then spiked into healthy donor (HD) EVs at varying concentrations ranging from 1.0E6 to 1.0E10 particles per mL to assess the calibration linearity and limit of detection (LOD) of the ILN assay. We observed good linearity within the diagnostic range, with an LOD of 1.0E6 and 4.0E7 of H82 cell‐derived EVs for DLL3 Exo‐mRNA and DLL3 tEV‐mProtein, respectively (Figure 1C,D). In comparison, the averaged EV DLL3 mRNA expression by qRT‐PCR exhibited good linearity but lower sensitivity, with an LOD of 5.0E7 of H82 cell‐derived EVs (Figure 1C). Similarly, using an ELISA kit, DLL3 mProtein expression was detectable only at a concentration of 1.0E11 particles per mL (Figure 1D). Overall, our ILN biochip assay demonstrated ≈50‐fold higher sensitivity than standard EV mRNA detection and 1000‐fold higher sensitivity than conventional protein detection methods.

### 2.2 Distinct EV Subpopulations for DLL3 mRNA and mProtein Detection

It is important to note that achieving complete purification of a specific EV subpopulation from biofluids‐ based on size, surface receptor, density, surface charge, or other characteristics‐ is challenging.^[^ ^41^, ^42^ ^]^ This is due to the overlapping sizes and contents of exosomes and microvesicles, which result from the dynamic nature of endocytic recycling, varying by cell type and physiological conditions.^[^ ^43^, ^44^, ^45^ ^]^

Tetraspanin proteins (CD63, CD81, and CD9) are highly expressed on the exosome surface^[^ ^46^, ^47^, ^48^ ^]^ and play key roles in multivesicular body and intralumenal vesicle (MVB/ILV) formation.^[^ ^49^ ^]^ To capture the exosome‐rich subpopulation (Exo) in EVs, we used biotin‐conjugated CD63, CD81 and CD9 antibodies. The best capture efficiency for DLL3 mRNA detection was obtained using a mixture of all three antibodies (Figure S3A, Supporting Information).

**Figure 2:**
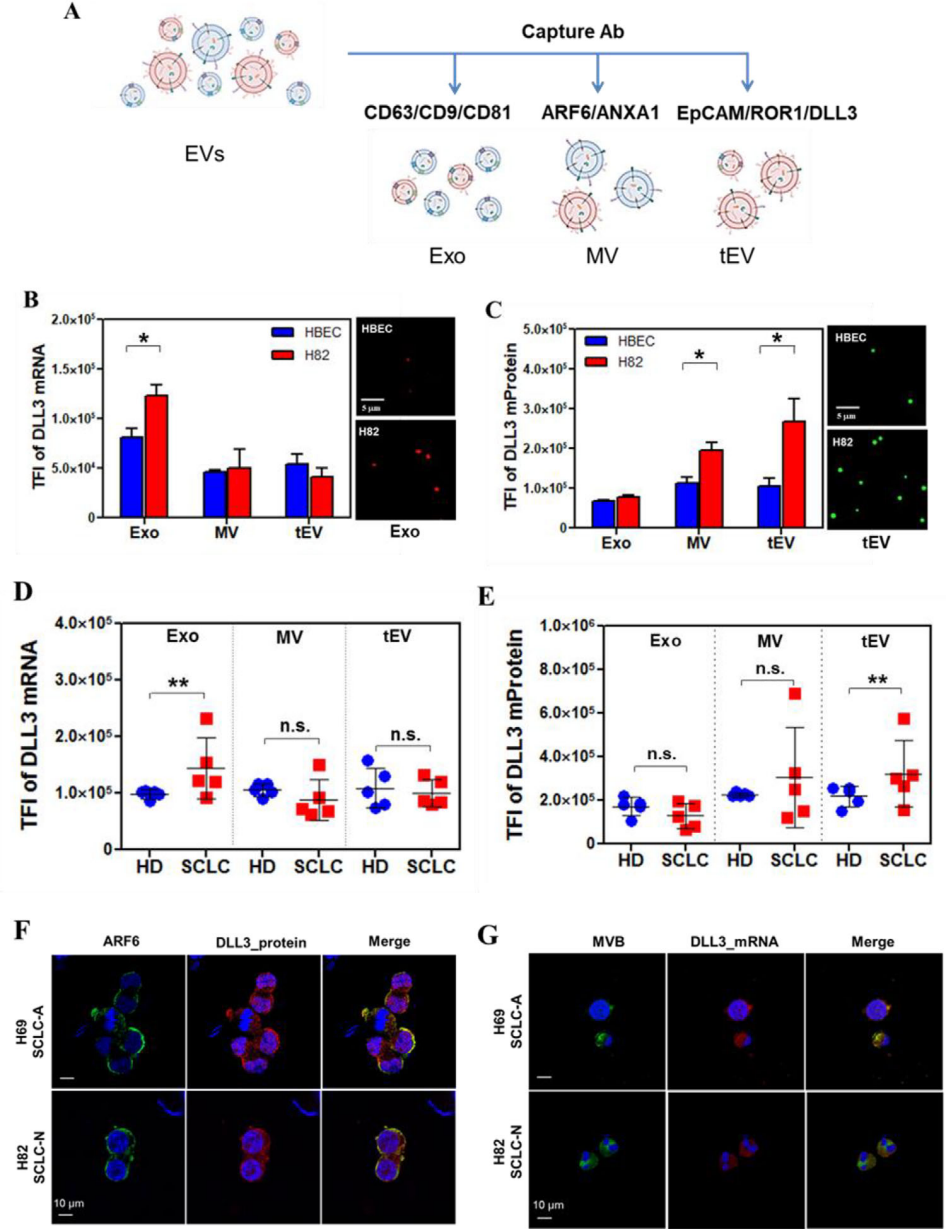
Distinct EV subpopulations for DLL3 mRNA and mProtein detection. A) Antibodies for capture different EV subpopulations (Exo, MV, and tEV). B,C) DLL3 mRNA and mProtein expression in the different EV subpopulations from H82 and HBEC cells. The small images represent a small surface area of one TIRF image. D,E) DLL3 mRNA and mProtein expression levels in EVs sorted by mixed capture antibodies from healthy donor (HD) and SCLC patient serum samples (*n* = 5). All data were presented as means (*n* = 2 wells, each well with 100 images). TFI; Total Fluorescence Intensity. *p‐*values were determined by the two‐way ANOVA test. *p < 0.05 and **p < 0.01. n.s: not significant. Co‐localization staining of F) DLL3 mRNA and late endosome (multivesicular body, MVB) and (G) DLL3 protein and ARF6 in SCLC‐A H69 and SCLC‐N H82 cell lines. Scale bar is 10 µm.

Since ADP‐ribosylation factor 6 (ARF6) and Annexin A1 (ANXA1) are known markers for cell membranes and microvesicles,^[^ ^50^, ^51^ ^]^ biotin‐conjugated ARF6 and Annexin A1 were used to capture microvesicle‐rich EV subpopulation (MV). For tumor‐associated EVs (tEV), we tested the capture efficiency of four cancer cell membrane‐specific antibodies: EGFR, EpCAM, ROR1, and DLL3.^[^ ^52^, ^53^, ^54^, ^55^ ^]^ Although EGFR is highly upregulated in many cancer types, it was inefficient for sorting SCLC EVs and quantifying DLL3 protein (Figure S3B, Supporting Information). In contrast, an equal‐weight mixture of DLL3, EpCAM, and ROR1 antibodies outperformed individual antibodies in capture efficiency (Figure S3B, Supporting Information). Therefore, we selected this antibody combination for isolating DLL3/EpCAM/ROR1^+^ tumor‐associated EVs (tEV), as it provides superior performance across human samples. A summary of the capture antibodies used for different EV subpopulations is shown in **Figure** **2**A.

We first assessed DLL3 mRNA and membrane protein expression in Exo, MV, and tEV subpopulations using EVs derived from SCLC cells (H82) and human bronchial epithelial cells (HBEC). Exo captured using a mixture of CD63/CD81/CD9 antibodies showed higher DLL3 mRNA expression in H82 cells but low expression in HBECs. This pattern was not observed in MV captured using the ARF6/Annexin A1 antibodies or tEV captured using DLL3/EpCAM/ROR1 antibodies, suggesting that DLL3 mRNA is less abundant in MV or tEV (Figure 2B). In agreement with this, RNA profiling data indicated that microvesicles contain less RNAs compared to exosomes.^[^ ^56^ ^]^

On the other hand, MV and tEV captured using DLL3/EpCAM/ROR1 antibodies exhibited higher DLL3 mProtein expression in H82 cells, but not in HBECs. In contrast, DLL3 mProtein expression in Exo captured using CD63/CD81/CD9 antibodies showed no significant differences between H82 and HBECs (Figure 2C).

Next, we tested EVs in serum samples from 5 HDs and 5 SCLC patients using the same antibody combinations. As shown in Figure 2D, DLL3 mRNA expression in Exo captured using CD63/CD81/CD9 antibodies was significantly higher in SCLC patients compared to HDs, whereas MV captured using ARF6/Annexin A1 antibodies and tEV captured using DLL3/EpCAM/ROR1 antibodies showed no significant differences. In contrast, DLL3 mProtein expression was low in Exo from both SCLC patients and healthy donors (HD), but MV and tEV analysis effectively distinguished SCLC patients from HD (Figure 2E). Since tEV vesicles were captured using monoclonal antibodies (mAbs) targeting multiple oncogenic proteins present on tumor cell membranes, it is not surprising that tEV outperformed MV as membrane protein‐based biomarkers. qRT‐PCR analysis of averaged DLL3 mRNA across all EV subpopulations showed no significant differences between SCLC patients, HD, and high‐risk smokers (HRS) control samples (Figure S4, Supporting Information). Taken together, proper selection of capture antibodies is critical for sorting EV subpopulations on the ILN biochip surface, which is essential for detecting mRNA and mProtein biomarkers.

To further investigate these findings, we performed cell staining for co‐localization in SCLC‐A H69 and SCLC‐N H82 cell lines. In both cells, DLL3 mRNA was strongly expressed in late endosomes (or MVBs), the intracellular origin of exosomes (Figure 2F), whereas DLL3 membrane protein expression was prominent on the cell membrane co‐localized with a major microvesice (MV) marker, ARF6 expression, where MV formed via outward budding (Figure 2G).

Additionally, we used size exclusion chromatography (SEC) with a qEV column (Figure S5A, Supporting Information) to fractionate EVs from H82 and H69 cells based on size (Figure S5B, Supporting Information). We found that larger EVs, enriched with microvesicle markers such as ANXA1 (Figure S5C, Supporting Information), exhibited stronger DLL3 protein expression (Figure S5D, Supporting Information), whereas smaller EVs, enriched with exosome markers such as CD63 (Figure S5E, Supporting Information), exhibited stronger DLL3 mRNA expression (Figure S5F, Supporting Information).

A similar trend is observed in other transcription factors and cancer types. For example, *Glypican 1* (GPC1) in pancreatic ductal adenocarcinoma (PDAC) cell lines and patient blood samples,^[^ ^57^ ^]^ and *Glypican 3* (GPC3) in hepatocellular carcinoma (HCC) patient blood samples (Figure S6, Supporting Information), follow the same pattern. Specifically, both GPC1 and GPC3 mRNA expressions are high in the exosome‐rich subpopulation but low in the microvesicle‐rich subpopulation, whereas both GPC1 and GPC3 membrane protein expressions are high in the microvesicle‐rich subpopulation but low in the exosome‐rich subpopulation.

This pattern is consistent with previous reports in the literature, where GPC1 membrane protein was found to be highly expressed on the tMV surface in pancreatic cancers,^[^ ^57^ ^]^ and plasma membrane receptors such as EGFR and EGFRvIII were upregulated in the MV fractions from cancer cells in glioma tumors.^[^ ^58^ ^]^

Together, our experimental results confirm that a higher DLL mRNA expression in the Exo subpopulation and a higher DLL3 mProtein expression in the tMV subpopulation for SCLC samples are reasonable.

### 2.3 Measurements of Subtype‐Specific Transcription Factors and DLL3 Expressions in SCLC Cell Lines and Patient Samples

We then investigated whether the EV‐based ILN assay could differentiate between molecular subtypes of SCLC and DLL3 expression. We collected EVs secreted by cell lines representing the three predominant SCLC subtypes: SCLC‐A with high ASCL1 (H69 and H209 cells), SCLC‐N with high NEUROD1 (H82 and H524 cells), and SCLC‐P with high POU2F3 (H526 and H1048 cells). For comparison, we also collected EVs secreted by a non‐small cell lung cancer cell line (A549) and human bronchial epithelial cells (HBEC) as negative controls, as these cells lack ASCL1, NEUROD1, and POU2F3 expression.

**Figure 3:**
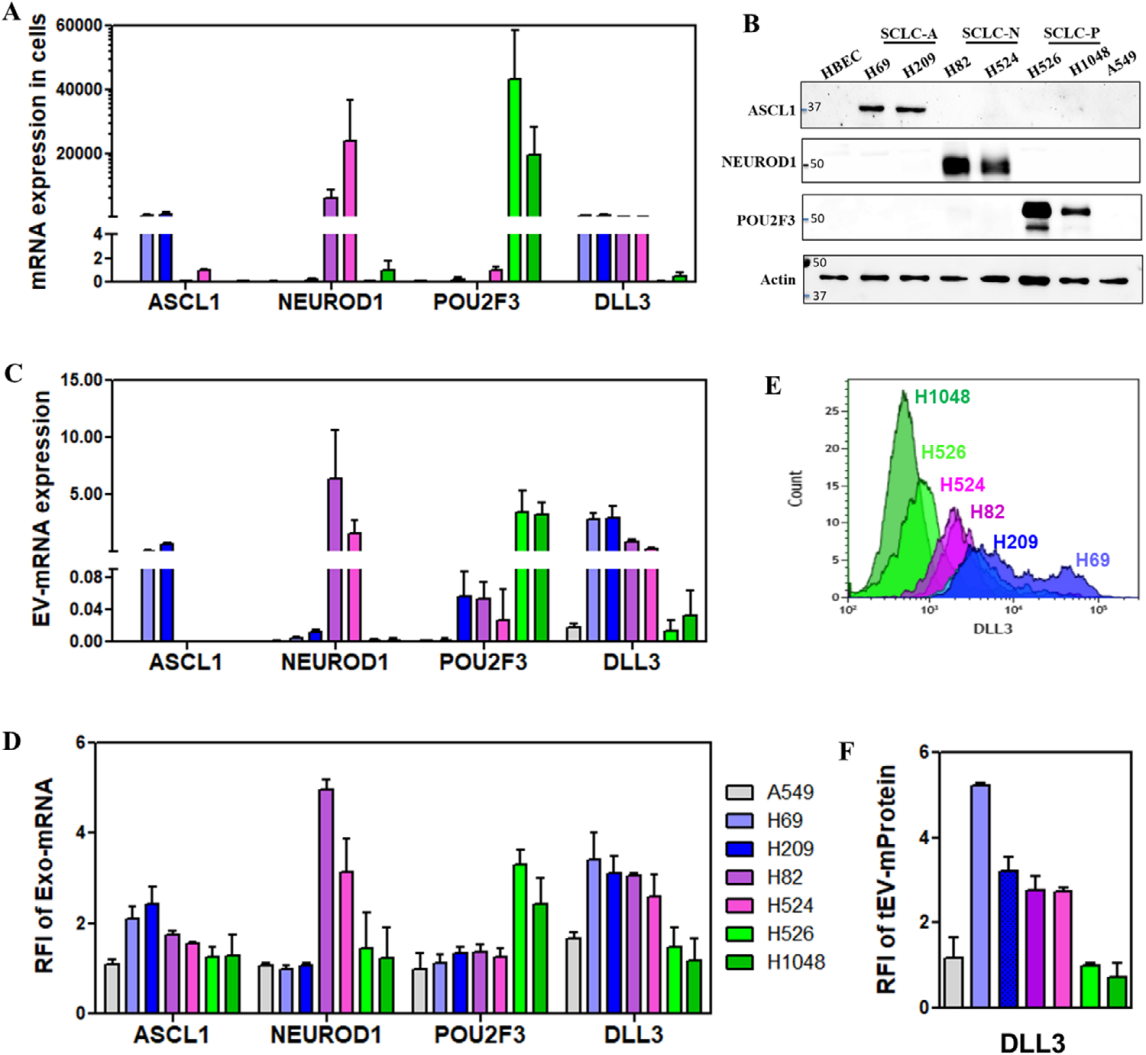
SCLC subtype transcription factors‐ ASCL1, NEUROD1 and POU2F3 mRNA, DLL3 mRNA, and DLL3 mProtein expressions in SCLC cells and SCLC cell secreted EVs. A) Subtype transcription factor and DLL3 mRNA expressions in SCLC cells by qRT‐PCR. B) Subtype transcription factor expressions in SCLC cells by Western blot. C) Subtype transcription factor and DLL3 mRNA expressions in SCLC cell secreted EVs by qRT‐PCR. D) Subtype transcription factor and DLL3 mRNA expressions in SCLC cell secreted EVs by ILN assay. E) DLL3 mProtein expression on SCLC cells by flow cytometry. F) DLL3 tEV‐mProtein expression in SCLC cell secreted EVs by ILN. RFI: Relative fluorescence intensity.

First, we measured cellular ASCL1, NEUROD1, and POU2F3 mRNA and protein expressions by qRT‐PCR (**Figure** **3**A) and Western blot (Figure 3B), respectively. As expected, A549 and HBEC cells showed little to no ASCL1, NEUROD1, or POU2F3 mRNA and protein expression. In contrast, H69 and H209 cells exhibited high ASCL1 expression (SCLC‐A), H82 and H524 cells had high NEUROD1 expression (SCLC‐N), and H526 and H1048 cells showed high POU2F3 expression (SCLC‐P) compared to A549 cells. EVs secreted from these cells followed a similar pattern, with high ASCL1, NEUROD1, and POU2F3 mRNA expressions detected by qRT‐PCR (Figure 3C).

Next, the ILN assay revealed that the Exo subpopulation from these cells contained high ASCL1 mRNA only in H69 and H209 cells, high NEUROD1 mRNA only in H82 and H524 cells, and high POU2F3 mRNA only in H526 and H1048 cells (Figure 3D). These findings suggest that ILN Exo‐mRNA profiling has the potential to identify distinct SCLC subtypes.

We then compared DLL3 expression in SCLC cells and SCLC cell‐derived EVs using qRT‐PCR. Both SCLC‐A (H69 and H209) and SCLC‐N (H82 and H524) cells exhibited high DLL3 mRNA expression compared to A549 cells, whereas SCLC‐P (H526 and H1048) cells exhibited low DLL3 mRNA expression (Figure 3A,C). Consistently, the ILN assay revealed that DLL3 Exo‐mRNA expression was significantly higher in both SCLC‐A and ‐N subtype cells than in SCLC‐P subtype cells and A549 cells (Figure 3D). Additionally, DLL3 mProtein expression in cells, as measured by flow cytometry (Figure 3E), followed the exact same trend as DLL3 tEV‐mProtein expression in cell‐derived EVs, as determined by the ILN assay (Figure 3F).

### 2.4 Exo‐mRNA Expression of Transcription Factors as Biomarkers for Clinical SCLC Subtyping

**Figure 4:**
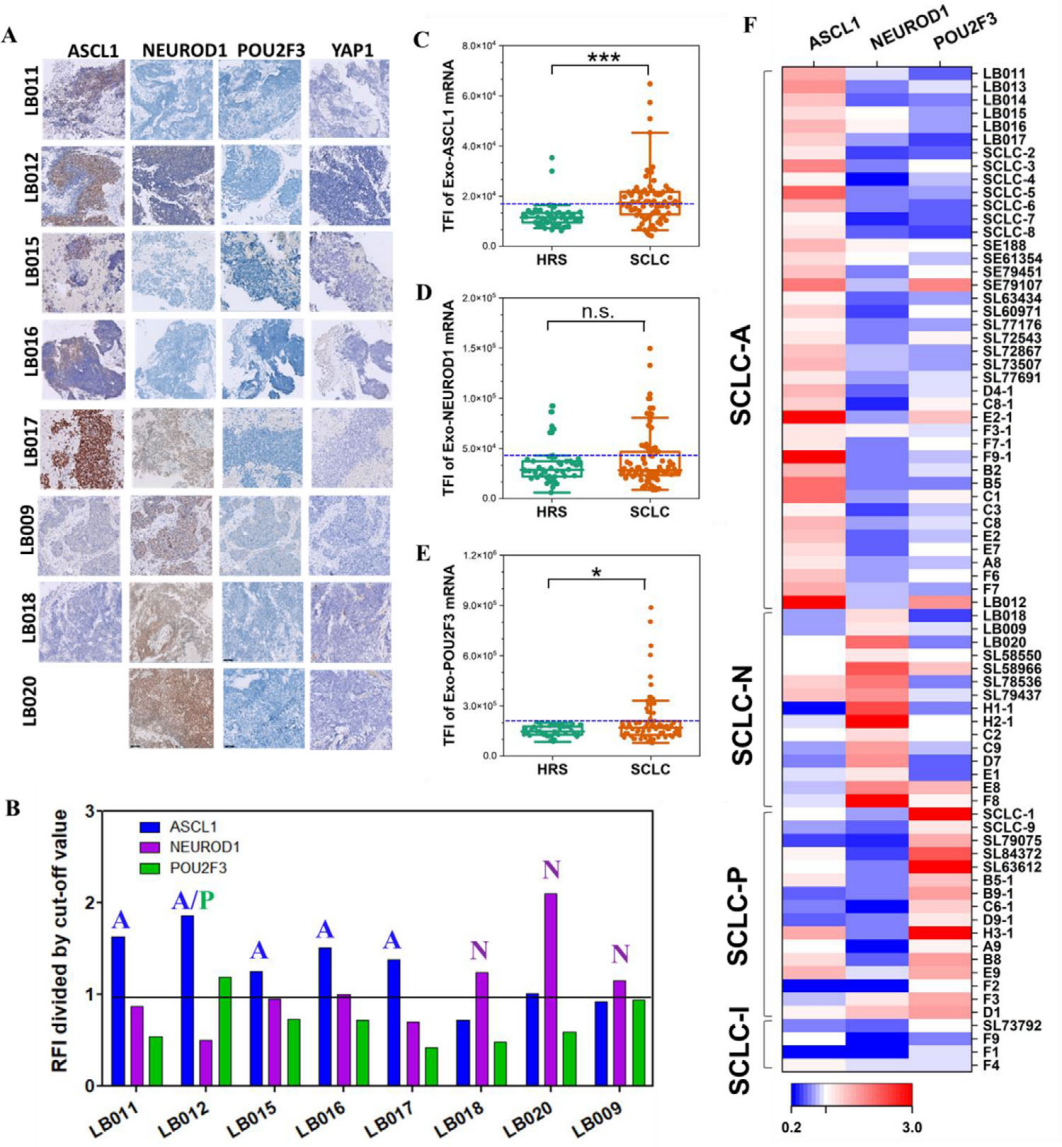
ILN biochip assay of subtype transcription factor expressions (Exo‐mRNA) for clinical SCLC patient samples. A) Representative tissue IHC images of SCLC subtypes. B) SCLC subtypes defined by the expressions of Exo‐mRNA of ASCL1, NEUROD1, and POU2F3 by ILN. C–E) ASCL1, NEUROD1 and POU2F3 Exo‐mRNA expressions in patients with SCLC compared to high risk smokers (HRS). F) Subtype heatmap of 76 SCLC patients by ILN assay. Patient samples starting with LB‐ and SCLC‐ were from TVGH and TMUH in Taiwan, respectively. Patient samples starting from SE and SL were from OSU. Others were from VCU. All data were presented as means (*n* = 2 wells, each well with 100 images). Pairwise comparison *p* values were determined by the Mann–Whitney U test. *p < 0.05 and ***p < 0.001. n.s.: not significant. Dotted lines in C indicate the cut‐off values from HRS (Blue dotted line in C–E).

Clinically, tumor tissue immunohistochemistry (IHC) staining is used to measure the protein expression of four transcription factors (ASCL1, NEUROD1, POU2F3, and YAP1) to identify SCLC subtypes. Here, we analyzed eight SCLC patients with available tissue IHC staining results and compared their subtypes with ILN analysis. Tissue IHC staining revealed 5 SCLC‐A and 3 SCLC‐N subtypes (**Figure** **4**A), and our ILN analysis consistently correlated with IHC staining results (Figure 4B). Together with the SCLC cell line results shown in Figure 3, these findings suggest that the ILN assay of EVs, as a liquid biopsy tool, can accurately reflect tissue expression for SCLC subtyping and diagnosis.

The cut‐off values shown in Figure 4B were determined by a total of 76 clinical SCLC patient samples from multiple institutions: Virginia Commonwealth University (VCU, *n* = 38 plasma) and the Ohio State University Cancer Hospital (OSU, *n* = 19 serum) in the US, as well as Taipei Veterans General Hospital (TVGH, *n* = 10 plasma) and Taipei Medical University Hospital (TMUH, *n* = 9 plasma) in Taiwan. A control group of high‐risk smokers (HRS) from VCU (*n* = 45 plasma) was included. The clinical characteristics of the patients are summarized in **Table** **1**.

**Table 1:** Clinical characteristic of SCLC patients.

Characteristics	HRS	SCLC			
Hospitals	VCU [*n* = 45]	VCU [*n* = 38]	OSU [*n* = 19]	TVGH [*n* = 10]	TMUH [*n* = 9]
Age (years) Median (range)	64.4 (52–74)	65 (43–85)	66 (57–82)	70 (54–82)	64.0 (57–81)
Gender, *n* (%) Male Female	23 (51.1%) 22 (48.9%)	20 (52.6%) 18 (47.4%)	10 (52.6%) 9 (47.3%)	8 (80.0%) 2 (20.0%)	7 (77.8%) 2 (22.3%)
Stage at diagnosis, *n* (%) IA/IB IIA/IIB III IV		8 (21.1%) 3 (7.9%) 7 (18.4%) 20 (52.6%)	2 (10.5%) 2 (10.5%) 5 (26.3%) 10 (52.6%)	‐ 1 (10.0%) 2 (20.0%) 7 (70.0%)	‐ 1 (11.1%) 2 (22.2%) 6 (66.7%)
Tobacco abuse, *n* (%) Smokers Non‐smokers	45 (100%) 0 (0%)	38 (100%) 0 (0%)	‐ ‐	8 (80.0%) 2 (20.0%)	9 (100%) 0 (0%)

HRS: high‐risk smoker.

ILN biochip analysis showed that ASCL1 Exo‐mRNA TFI values were significantly higher in SCLC patients compared to HRS (***p < 0.001) (Figure 4C). However, NEUROD1 Exo‐mRNA levels showed no difference between SCLC and HRS (Figure 4D), while POU2F3 Exo‐mRNA exhibited a modest but significant difference between SCLC patients and HRS (*p < 0.05) (Figure 4E).

Distinct cut‐off values for each transcription factor were established based on the highest TFI of HRS after excluding outliers from the Whisker box plot: ASCL1 Exo‐mRNA (TFI = 14 295), NEUROD1 Exo‐mRNA (TFI = 42 819), and POU2F3 Exo‐mRNA (TFI = 185 157). The TFI of each SCLC patient was then divided by the cut‐off value of the corresponding transcription factor. Patients were classified as SCLC‐A, ‐N, and ‐P subtypes if their TFI_patient_/TFI_cutoff_ ratio exceeded 1. The subtyping results for all 76 SCLC patients, as determined by the ILN analysis, are summarized in a heatmap (Figure 4F), while the proportion of each subtype is presented in Figure S7 (Supporting Information). The distribution of SCLC subtypes: 53.9% SCLC‐A, 19.7% SCLC‐N, 21.1% SCLC‐P, and 5.3% SCLC‐I, closely matches the proportion reported in the literature based on IHC staining of tumor tissue samples.^[^ ^7^, ^8^ ^]^

### 2.5 Exo‐mRNA and tEV‐mProtein Expression of DLL3 as Biomarkers for Clinical SCLC Diagnosis

**Figure 5:**
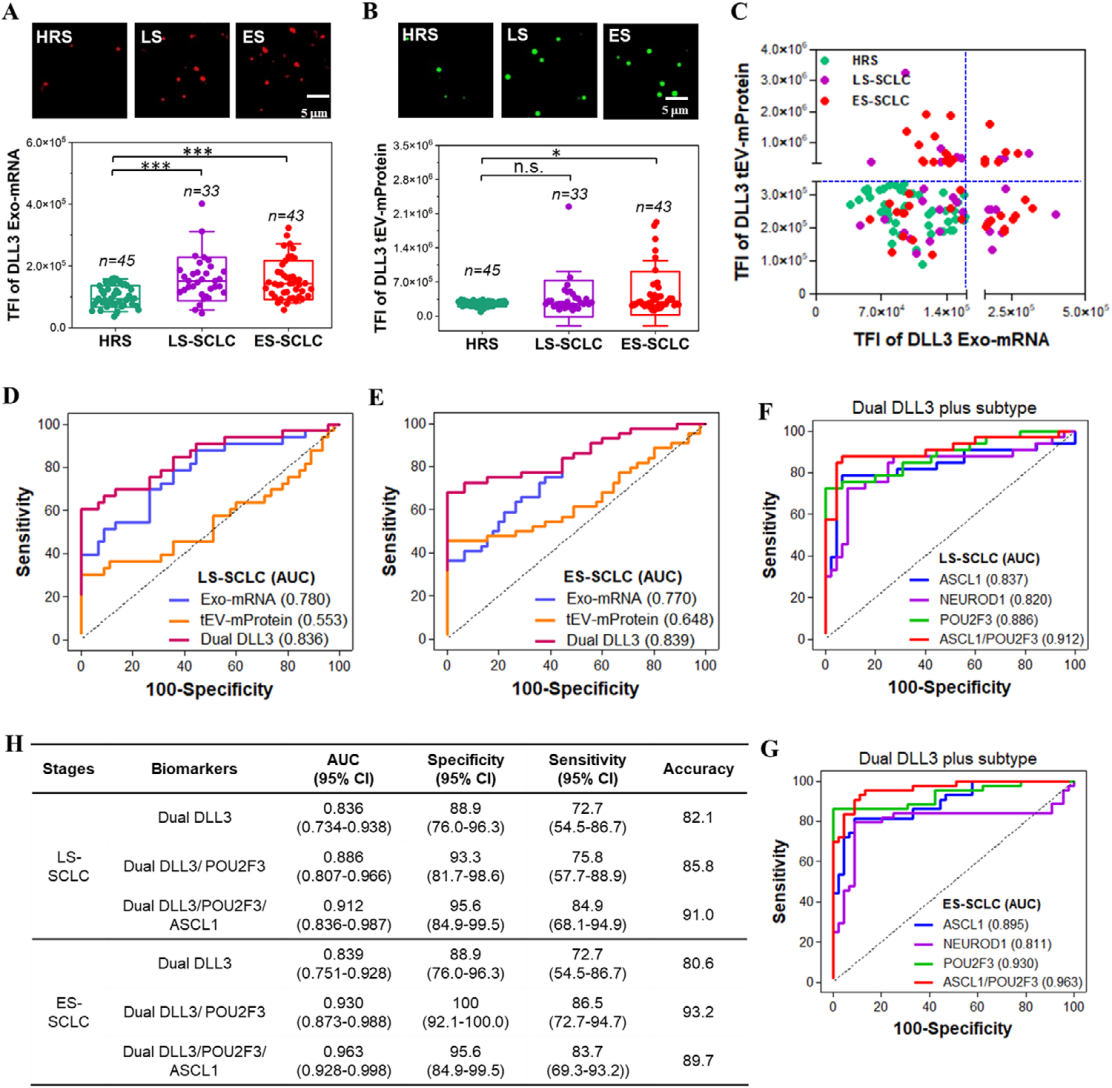
ILN biochip analysis of DLL3 Exo‐mRNA, DLL3 tEV‐mProtein, and transcription factor Exo‐mRNA expressions for SCLC diagnosis. A,B) DLL3 Exo‐mRNA and DLL3 tEV‐mProtein expressions and their representative TIRF images in patients with Limited Stage (LS) and Extended Stage (ES) SCLC patients compared to high‐risk smokers (HRS). C) Scatter plot of DLL3 Exo‐mRNA and tEV‐mProtein as a dual‐biomarker. D,E) AUC/ROC curves of DLL3 Exo‐mRNA and tEV‐mProtein expressions as a single‐ or dual‐biomarker in patients with LS‐ and ES‐SCLC compared to HRS. F,G) DLL3 Exo‐mRNA and tEV‐mProtein expression, incorporated with Exo‐mRNA expression of subtype transcription factors as biomarker classifiers for LS‐ and ES‐SCLC patients compared to HRS. H) Analysis of AUC, specificity, sensitivity and diagnostic accuracy. AUC, area under the curve. ROC, receiver operating characteristic. Pairwise comparison *p* values were determined by the Mann–Whitney U test. *p < 0.05 and ***p < 0.001. All data were presented as means (*n* = 2 wells, each well with 100 images).

To evaluate the potential of Exo‐mRNA and tEV‐mProtein expression as a biomarker for SCLC diagnosis, we assessed DLL3 expression in the 76 clinical SCLC patient and 45 HRS samples listed in Table 1. SCLC is classified into limited‐stage (LS) and extensive‐stage (ES), where LS includes stages I‐III patients and ES includes stage IV patients. The ILN biochip assay revealed significantly higher total fluorescence intensity (TFI) values for DLL3 Exo‐mRNA in both LS and ES patients compared to HRS (***p < 0.001) (**Figure** **5**A). Additionally, DLL3 tEV‐mProtein expression was significantly higher in ES‐SCLC patients (*p < 0.05) compared to HRS (Figure 5B).

Given the substantial overlap in EV DLL3 expression between SCLC patients and HRS, we conducted a combined analysis of DLL3 Exo‐mRNA and tEV‐mProtein expression as a dual‐biomarker for SCLC detection. As shown in Figure 5C, all HRS samples exhibited both low Exo‐mRNA and low tEV‐mProtein expressions of DLL3 (i.e., L/L) in plasma. In contrast, SCLC patients displayed one of the following DLL3 expression patterns: H/H‐ high Exo‐mRNA and high tEV‐mProtein, H/L‐ high Exo‐mRNA and low tEV‐mProtein, or L/H‐ low Exo‐mRNA and high tEV‐mProtein. The receiver operating characteristic (ROC) analysis was conducted to assess the diagnostic performance of these biomarkers. The area under the curve (AUC) values for LS (Figure 5D) and ES (Figure 5E) compared to HRS were: DLL3 Exo‐mRNA: 0.780 (LS) and 0.770 (ES), tEV‐mProtein: 0.553 (LS) and 0.648 (ES), and dual Exo‐mRNA/tEV‐mProtein: 0.836 (LS) and 0.839 (ES). These results suggest that the combination of Exo‐mRNA and tEV‐mProtein expression enhances the diagnostic accuracy of SCLC detection.

To differentiate SCLC patients from HRS controls in a clinical setting, distinct cut‐off values of Exo‐mRNA (TFI = 160 238) and tEV‐mProtein (TFI = 336 847) shown in Figure 3C were established using ROC curves from SCLC clinical samples with 100% specificity. The cut‐off values can be converted into analytical quantities based on the calibration curves shown in Figure 1C,D: DLL3 Exo‐mRNA: 1.60E7 H82 EVs/mL HD serum, and tEV‐mProtein: 6.0E8 H82 EVs/mL HD serum. These values provide a quantifiable reference for SCLC detection in clinical diagnostics.

### 2.6 EV DLL3 and Subtype Transcription Factors as Enhanced Biomarkers for Clinical SCLC Diagnosis

To further enhance the diagnostic accuracy, we incorporated Exo‐mRNA expression of subtype transcription factors (ASCL1, POU2F3, and NEUROD1) into the dual DLL3 Exo‐mRNA/tEV‐mProtein biomarker. When ASCL1 or POU2F3 Exo‐mRNAs was individually combined with the dual DLL3 Exo‐mRNA/tEV‐mProtein biomarker, the AUC/ROC improved: LS‐SCLC patients from 0.836 to 0.837 with ASCL1, and from 0.836 to 0.886 with POU2F3; ES‐SCLC patients from 0.839 to 0.895 with ASCL1, and from 0.839 to 0.930 with POU2F3 (Figure 5F,G). However, adding NEUROD1 Exo‐mRNA to the dual DLL3 Exo‐mRNA/tEV‐mProtein biomarker slightly decreased the AUC/ROC: LS‐SCLC patients from 0.836 to 0.820, and ES‐SCLC patients from 0.839 to 0.811. The reduction may be attributed to relatively high NEUROD1 Exo‐mRNA expression in some HRS samples (Figure 5F,G).

The highest AUC/ROC was observed when ASCL1 and POU2F3 Exo‐mRNA expressions were combined with the dual DLL3 Exo‐mRNA/tEV‐mProtein expression for a biomarker classifier: 0.912 for LS‐SCLC patients and 0.963 for ES‐SCLC patients (Figure 5F,G). A comprehensive analysis for specificity, sensitivity, and diagnostic accuracy is summarized in Figure 5H.

As a diagnostic biomarker, ASCL1 Exo‐mRNA alone was able to distinguish ES‐SCLC from HRS (AUC = 0.809) but was not effective for LS‐SCLC (AUC = 0.694) (Figure S8A,B, Supporting Information). In contrast, NEUROD1 and POU2F3 Exo‐mRNA alone did not demonstrate significant biomarker potential (Figure S8A,B, Supporting Information). Combining ASCL1 and POU2F3 Exo‐mRNA slightly increased the AUC of ROC to 0.818 for ES‐SCLC, but it remained insufficient for LS‐SCLC diagnosis (Figure S8C,D, Supporting Information).

## 3 Discussion

Using an ILN biochip assay with proper antibodies, we sorted exosome‐rich (Exo) subpopulations using CD63/CD9/CD81 antibodies, microvesicle‐rich (MV) subpopulations using ARF6/Anexin A1 antibodies, and tumor‐associated EVs (tEV) using SCLC specific membrane proteins such as DLL3, EpCAM and ROR1 antibodies. We demonstrated that the dual DLL3 Exo‐mRNA/tEV‐mProtein biomarker can distinguish 80% of SCLC patients from high‐risk smokers. However, DLL3 expression is low in some SCLC patients with high non‐NE tumor cells. Within non‐NE SCLC cells, 75% expresses POU2F3.^[^ ^59^ ^]^ Additionally, ≈17% of SCLC patients with low DLL3 expression exhibit high ASCL1 expression.^[^ ^60^ ^]^ By incorporating EV transcription factors for SCLC subtypes into the DLL3‐based biomarker classifier, our ILN biochip assay can detect more than 90% of LS‐ and ES‐SCLC patients and accurately determine their subtypes.

Microvesicles are formed through outward budding of the cell membrane, making the microvesicle‐rich EV subpopulation most suitable for mProtein‐based EV biomarkers. In contrast, mRNAs are transcribed by DNA in the cell nucleus. Once mRNAs diffuse out from the nucleus, they bind to ribosomes for protein translation. Fragments of mRNAs that have been used for protein translation are degraded by endosomes in the cytosol and some eventually end up in exosomes. Additionally, excess mRNAs, such as upregulated transcription factors in cancer cells, are encapsulated in stress granules (SGs) for storage via RNA‐binding proteins. These lipid‐free SGs may “hitchhike” on lipid‐based endosomes to facilitate intracellular mRNA transport.^[^ ^61^, ^62^, ^63^, ^64^ ^]^ Some mRNA in SGs may move into ILVs in MVBs via SG‐endosome fusion. Since ILVs in MVBs are precursors to exosomes, it is not surprising that upregulated mRNAs in cancer cells are abundant in the exosome‐like EV subpopulation, while few mRNAs are found in the microvesicle‐like EV subpopulation.^[^ ^65^ ^]^ The biogenesis of mRNA in the exosome‐rich EV subpopulation is a complex phenomenon and more detailed investigation is warranted to gain a better understanding of the mechanism.

It should be noticed that different EV subpopulations overlap in terms of size and surface receptors. It is essentially impossible to sort EVs into pure exosome and microvesicle populations in any biofluids. The goal of this study is to develop an easy‐to‐use EV‐based liquid biopsy technique that can accurately distinguish patients from non‐patients in disease diagnosis, not to conduct an EV characterization study. Although tetraspanin proteins such as CD63, CD9, and CD81 are essential for ILV (the precursor of exosomes) formation in MVBs and are rich on exosome surface, they may also express on the microvesicle surface. Therefore, EVs captured by a mixture of CD63/CD9/CD81 antibodies on the ILN biochip surface merely represent an EV population rich in exosomes, but not pure exosomes. Exo represents an exosome‐rich EV population, but not a pure exosome population. Similarly, MV represents an EV population rich in microvesicles, but not pure microvesicles, and tEV represents an EV population rich in tumor‐associated EVs, but not pure tumor EVs. Our results demonstrate that it is unnecessary to capture pure exosomes or cancer cell‐secreted microvesicles in biofluids to achieve highly accurate cancer diagnosis.

The poor efficacy of current therapeutic options in SCLC may be partially attributable to the recently recognized high molecular heterogeneity of SCLC cells. If blood‐based liquid biopsy can offer reliable biomarkers for diagnosis, prognosis, and surveillance, there is potential for the development of improved strategies for early detection and therapeutics that achieve precision medicine in subtype‐based SCLC therapy. Our ILN assay can easily facilitate frequent real‐time tracking of DLL3 and other transcription factors throughout therapy which could inform surveillance. Further, this capability should be valuable for the development and implementation of anti‐DLL3‐based immunotherapy in SCLC.

## 4 Experimental Section

### Cell Lines, Antibodies, and Molecular Beacons

SCLC cells (H69, H209, H82, H524, and H526) maintained in RPMI 1640 medium supplemented with 10% fetal bovine serum (FBS), 100 U mL^−1^ of penicillin and 100 µg mL^−1^. SCLC cells (H1048) maintained in DMEM:F12 medium supplemented with 10% FBS, 100 U mL^−1^ of penicillin, and 100 µg mL^−1^ of streptomycin. NSCLC cells (A549) were maintained in DMEM medium supplemented with 10% FBS, 100 U mL^−1^ of penicillin, and 100 µg mL^−1^ of streptomycin. Non‐cancerous cells (HBEC) were maintained in BronchiaLife cell culture medium. All cells were cultured at 37 °C in a 5% CO_2_/95% air humidified atmosphere. For capture antibodies, biotin anti‐CD63 antibody (ab1334331), biotin anti‐CD9 antibody (ab28094), and biotin anti‐CD81 antibody (ab239238) were purchased from Abcam (Cambridge, MA). Anti‐EpCAM antibody (MAB960, R&D system), anti‐ROR1 antibody (357816, BioLegend), anti‐DLL3 antibody (MAB45191, R&D systems), anti‐ARF6 antibody (NBP2‐41263, Novus Biologicals) and anti‐Annexin A1 antibody (AF3770, R&D systems) were purchased and biotinylated using EZ‐Link micro sulfo‐NHS biotinylation kit (#21925, ThermoFisher) according to the manufacturers’ instructions. Human DLL3 PE‐conjugated antibody (FAB4315P, R&D system) was purchased and used for EV protein detection. Molecular beacons that detected DLL3, ASCL1, NEUROD1, and POU2F3 mRNAs were custom‐synthesized by Integrated DNA Technologies, Inc. (San Diego, California). Sequences were 5′‐ +GCA +GCT /iCy3/ +CGA +AGA +CGC CAG CGA AGG CGT CTT CG /3BHQ_2/‐3′ for DLL3 MB, 5′‐ C+CA A+CG /iCy3/+CCA +CTG A+CA A+GA AAG CAC TCT TGT CAG TGG /3BHQ_2/‐3′ for ASCL1 MB, 5′‐+CTC +GCT/iCy3/+CAT +GAT GTG AAT GGC GCC ATT CAC A/3BHQ_2/‐3′ for NEUROD1 MB, and 5′‐ CTC+TGC/iCy3/+TTG +AAG +GTC TTG GCA TGC CAA GAC CT/3BHQ_2/‐3′ for POU2F3.

### Biochip Fabrication

A cleaned high‐precision glass coverslip (D263 M Glass, 24 × 75 nm rectangle, 0.15 mm thickness, Schott AG, Germany) was first activated using a UV‐ozone cleaner for 15 min. Thin layers of 2‐nm thick Ti and 12‐nm thick Au were sequentially deposited using a Denton‐e‐beam evaporator (DV‐502A, Moorestown, NJ). The Ti/Au‐coated glass was immersed into a linker solution composed with 1‐thiahexa(ethyleneoxide) lipidic anchor molecule WC14 [20‐tetradecyloxy‐3,6,7,12,15,18, 22‐heptaoxahexa‐tricontane‐1‐thiol], a lateral spacer β‐mercaptoethanol (β‐ME), and biotin‐PEG‐SH (molar ratio = 15:83:2) for overnight at RT. The coverslip was then washed three times with ethanol and dried with liquid nitrogen air. Then, the glass coverslip was attached to a 64‐well chamber (Grace Bio‐Labs ProPlate multiwell chamber, Sigma‐Aldrich) and washed thoroughly with PBS. Next, 0.005% (w/v) neutravidin‐conjugated gold nanoparticles (40 nm, Nanopartz Inc.) in PBS were applied into each well of biochip for 2 h at RT on a shaker. After rinsing six times with PBS, the biochip was incubated with capture antibody mixture (20 µg mL^−1^ total) for overnight at 4 °C. After the antibodies were tethered onto the nanogold surface, the biochip was washed six times with PBS, and then blocked with 4% (w/v) BSA in PBS for 1 h at RT. After washed with PBS, EVs isolated from SCLC patient serum/plasma or cell culture supernatant were applied into each well of the biochip for 2 h at RT.

### EV Isolation from Cell Culture

Cells were grown for 48 h in culture medium supplemented with 2% EV‐depleted fetal bovine serum (A272081, ThermoFisher). Conditioned culture medium (50 mL) was collected and centrifuged at 2500×g for 30 min to remove cell debris. The supernatants were concentrated to 1 mL using Amicon Ultra centrifugal units (10 kDa MWCO, Fisher Scientific) at 2500x g. To purify EVs, 0.5 mL was loaded onto a size exclusion qEV_70_ column and started to collect fractions immediately according to the manufacturers’ instructions. Fractions 6–12 were combined and concentrated to 100 µL using Amicon Ultra‐4 centrifugal filters with 10 kDa MWCO. EVs were quantified using NanoSight nanoparticle tracking analysis.

### EV Isolation from Human Serum/Plasma Samples

Blood samples were collected with the informed consent of the patients and high‐risk smokers, and the study was performed with the approval of the Institutional Review Boards at OSU (Protocol # 2008C0093) and Virginia Commonwealth University (Protocol #HM2471) in the US, Taipei Veterans General Hospital (Protocol # 201701016C) and Taipei Medical University Hospital (Protocol #N201604036) in Taiwan. Blood samples from SCLC patients were collected at diagnosis and serum/plasma was isolated by centrifugation. Healthy donor serum samples were purchased from Zen‐bio, Inc. EVs from serum, plasma, or cell culture medium were isolated using the Plasma Exosome Isolation Kit from Invitrogen with slight modifications. Briefly, 150 µL of serum or plasma was initially treated with 5 µL of proteinase K (PK) for 10 min at room temperature, followed by the addition of 50 µL of TEI solution to precipitate EVs. After centrifugation, the precipitate was resuspended in 150 µL of PBS and then stored at −80 °C until use.

### Calibration of DLL3 Exo‐mRNA and tEV‐mProtein using Cancer cell EVs

EVs from H82 cells were purified using qEV_70_ column and spiked into HD EVs at different concentrations ranging from 4.0E7 to 4.0E10 EVs/mL, while the healthy donor EVs were kept constant. After four‐fold dilution of each sample, the DLL3 Exo‐mRNA and DLL3 tEV‐mProtein levels were measured using ILN biochip assay. Calibration experiments were repeated three times.

### EV DLL3 mRNA Detection by ILN Assay

Cationic lipid nanoparticles (CLN) encapsulated with the DLL3 molecular beacon (MB) were synthesized and used to determine DLL3 mRNA expression. Lipids stock solution in ethanol was prepared using 1,2‐dioleoyl‐3‐tromethylammonium‐propane (DOTAP, Avanti Polar Lipids), 1,2‐dioleoyl‐sn‐glycero‐3‐phosphocholine (DOPC, Avanti Polar Lipids), cholesterol and 1,2‐distearoyl‐sn‐glycero‐3‐phosphoethanolamine‐N‐[biotinyl(polyethyleneglycol)2000] (DSPE‐PEG2000‐biotin, Avanti Polar Lipids) at a molar ratio of 50/33/15/2. To synthesize CLN‐MBs, lipid stock solution was mixed with MB and scramble (Cel‐miR‐54) solution at a molar ratio of 1:20. After sonication, the mixture was injected into PBS and further sonicated for 1 min at RT. The CLN‐MB was then dialyzed against PBS buffer at RT for 2 h using an MWCO 20 kDa dialysis device to remove residual free MB and scramble oligonucleotide. The particle size and zeta potential (ζ) of CLN‐MB was determined using dynamic light scattering and ZetaPALS from Brookhaven Instruments Corp. (Worcestershire, NY). After the biochip was incubated with EVs, CLN‐MB was added to the wells and incubated for 1 h at 37 °C. Then, the biochip was washed with PBS and the MB fluorescence signals were determined using TIRF microscopy (Nikon Eclipse Ti Inverted Microscope System).

### EV DLL3 Membrane Protein Detection

The biochip was incubated with EVs and then blocked with 4% (w/v) BSA solution for 1 h at room temperature. Then human DLL3 PE‐conjugated antibody (1:500 dilutions) in 1% (w/v) BSA PBS solution was added to each well on the biochip and incubated for 1 h at RT. The biochip was washed with PBS and the fluorescence signals were determined using TIRF microscopy.

### TIRF Measurements and Image Analysis

TIRF microscopy was used to record sample images. For mRNA and mProtein detection, a 50 mW 561 nm laser at 40–5% power was used to excite Cy‐3‐labeled MB and PE‐labeled antibodies. 100 (10 × 10 array) images were collected using an Andor iXon EMCCD camera with a 100× oil immersion lens and 200 ms exposure time. Images were analyzed with MATLAB software (R2019B) using custom code. An automatic algorithm was used to identify all bright spots of the apparent signal in each TIRF image. The background noise was removed by a Wavelet de‐noising method, and the net signal for all bright signals was obtained. The net fluorescence intensity of each spot was then calculated by subtracting the mean intensity of pixels in the spot from the mean intensity of pixels surrounding the spot. Proper cutoff was employed based on the spot size.^[^ ^57^ ^]^

### Western Blot

Total protein from SCLC cells (H69, H209, H82, H524, H526, and H1048) were extracted by RIPA lysis buffer and incubated at 95 °C for 5 min. Proteins were electrophoretically separated by SDS‐PAGE and then transferred onto a polyvinylidene difluoride (PVDF) membrane. After blocking with 5% non‐fat dry milk overnight, the membranes were incubated with primary antibodies at 4 °C for 16 h and then horseradish peroxidase (HRP)‐conjugated secondary goat anti‐rabbit or anti‐mouse antibody at 37 °C for 1 h. Immunodetection was performed and quantified by chemiluminescence (ECL) reagents (ThermoFisher Scientific) and Image Studio Lite V5.2 (Licor Biosciences).

### Flow Cytometry

SCLC cells (H69, H209, H82, H524, H526 and H1048) were stained using fluorescence‐labeled human DLL3 monoclonal antibodies (ab310446). Flow cytometric experiments were performed by using Beckman Coulter Gallios flow cytometers (Brea, CA). Absolute cell concentrations were obtained by quantitative flow cytometry using Count‐Bright absolute counting beads (Invitrogen).

### qRT‐PCR

RNA from cells or EV pellets or H82 spiked EVs was extracted by total RNA purification kits (Norgen Biotek) according to the manufacturer's instructions. Complementary DNA (cDNA) was generated by a high‐capacity cDNA reverse transcription kit (Cat#: 4368814, Thermo Fisher Scientific) from extracted RNA. Subsequently, mRNA expression for DLL3 (Hs01085093_m1), ASCL1 (Hs00269932_m1), NEUROD1 (Hs001594598_m1), POU2F3 (Hs00205009_m1) and GAPDH (Hs02786624_g1) was quantified using a TaqMan Gene Expression assay (Thermo Fisher Scientific) on a real‐time PCR instrument (Applied Biosystems).

### ELISA

EVs from H82 cells were spiked in healthy donor serum EVs at concentrations ranging from 1.0E6 to 5.0E10 particles per mL while maintaining the serum‐derived EV concentration at 1.0E11 particles per mL. One hundred µL of EV samples were added to the ELISA plate and their DLL3 protein expressions were detected according to the manufacturer's instructions.

### Immunofluorescence and Fluorescence In Situ Hybridization

The two DLL3‐expressing cell lines, NCI‐H69 and NCI‐H82 (1.0E5 per well, ATCC), were seeded onto glass slides in low‐serum RPMI 1640 adhesion medium for 24 h. The SCLC cells were then fixed with 4% PFA for 10 min, followed by two PBS washes on ice. DLL3 protein was detected using fluorescence‐labeled anti‐DLL3 antibodies (NBP3‐28317, Novus Biologicals, CO, USA), while DLL3 mRNA was visualized using fluorescence in situ hybridization (FISH) probes. The FISH probes were designed using the Qiagen ISH database. Probe sequences for DLL3 mRNA were AGAGTGGATCTGCAGCTCGAAP for positions 116–136, TGGAAGGAGCAGATATGACAT for positions 1752–1772, and AGAGAAGATGGCAGGTAGCTCA for positions 1969–1990. Late endosomal multivesicular bodies (MVBs) were stained with fluorescence‐labeled anti‐Rab7 (17286S, Cell Signaling, CO, USA), while cytosolic macrovesicular precursors were labeled with fluorescence‐labeled anti‐ARF6 (NBP2‐41263, Novus Biologicals, CO, USA). Colocalization was analyzed using confocal microscopy (Zeiss LSM 900, Germany).

### Statistical Analysis

The MATLAB R2019a was used for TIRF image analysis. To compare the difference in between groups, a Mann–Whitney U test was used. ROC curves were used to determine the sensitivity and specificity to compare AUCs of serum/plasma from SCLC patients and control of high‐risk smokers. All clinical samples were measured in two wells on a biochip. *p*‐values for pairwise comparisons were obtained using a two‐tailed Student's *t*‐test. The data were expressed as mean ± SD. For the prognosis study, patients were categorized into two groups according to the DLL3 expression cut‐off. Differences in progression‐free survival between these two groups were assessed through Kaplan–Meier survival curves and the log‐rank test.

## Conflict of Interest

L.J.L. is a shareholder at Spot Biosystems Ltd. Other co‐authors declare no competing interests.

## Author Contributions

H.L. and C.L.C. contributed equally to this work. L.J.L., S.P.N., C‐Lu.C., C‐Y.F.H., and C‐Li. C. designed the study. S.P.N., C‐Lu.C., H‐L.L., D.C., G.R., M.S., and R.T. provided patient samples. S.P.N., G.R., and M.S. provided SCLC cell lines, L.J.L., E.R., H.L., and K.J.K. developed the ILN technology. H.L. and Y.C. performed ILN assay. C‐Li.C., B‐D.H., G‐W.L., and H.L. performed cell line culture and cell/EV characterization experiments. K.J.K. and H.L. optimized and fabricated ILN biochip and designed molecular beacons. X.W. developed the MATLAB code for image analysis. H.L. and X.M. performed statistical data analysis. H.L., T‐S.C., and Y‐S.C. performed EV purification using total exosome isolation kit. X.Y.R drew the schematic figures. J.P., Y.M., and T.N.K. assisted cell culture, NTA analysis, and EV purification using qEV column. H.L., L.J.L., and C‐Li.C. wrote the paper with feedback from C‐L.C., S.P.N, C‐Y.F.H., D.C., and C.C.

## Supporting information



Supporting Information

## Data Availability

The data that support the findings of this study are available from the corresponding author upon reasonable request.
