# Supplementary material for: Extracellular Vesicular Delta‐Like Ligand 3 and Subtype Transcription Factors for Small Cell Lung Cancer Diagnosis
Source: Adv Sci (Weinh). 2025 Apr 26;12(22):2416711. doi: 10.1002/advs.202416711 (PMC12165112; doi:10.1002/advs.202416711)
Supplement: Supplementary file 1 — Supporting Information [file ADVS-12-2416711-s001.docx]

Supplementary Materials for

**Extracellular Vesicular Delta-Like Ligand 3 and Subtype Transcription Factors for Small Cell Lung Cancer Diagnosis**

Hong Li *et al.*

*Co-corresponding authors: [clchiang@nycu.edu.tw](mailto:clchiang@nycu.edu.tw); [cyhuang5@nycu.edu.tw](mailto:cyhuang5@nycu.edu.tw); [clchiang@vghtpe.gov.tw](mailto:clchiang@vghtpe.gov.tw); [Patrick.Nana-Sinkam@vcuhealth.org](mailto:Patrick.Nana-Sinkam@vcuhealth.org); [lee.31@osu.edu](mailto:lee.31@osu.edu)

**This file includes:**

Figure S1. EV concentration measured by tunable resistive pulse sensing.

Figure S2. Images of the ILN biochip surface with saturated EVs.

Figure S3. DLL3 mRNA and mProtein expression in EVs sorted by individual and mixed capture antibodies from a healthy donor (HD) and two SCLC patient (SCLC-1, SCLC-2) serum samples.

Figure S4. Comparison of ILN assay and qRT-PCR for EV DLL3 mRNA expression in SCLC and non-patient serum samples.

Figure S5. EV markers, DLL3 mRNA, and mProtein levels in large and small EVs isolated using size exclusion chromatography (SEC) with a qEV column.

Figure S6. GPC3 mRNA and mProtein expressions in different EV subpopulations from HCC (*n* = 5) and HD (*n* = 5) samples.

Figure S7. Estimates of relative frequencies of the four subtypes based on ILN assay with 95% CIs (*n* = 76).

Figure S8. AUC/ROC curves of subtype transcription factors in SCLC patients with limited-stage and extensive-stage as single biomarker or combined biomarker.

**Methods**

**EV isolation using qEV column**

EVs from H82 and H69 cell supernatant were fractionized using size exclusion qEV_70_ columns. qEV column was washed and equilibrated with PBS before use. Briefly, 0.5 mL cell culture supernatants were loaded onto the column and start collecting fractions immediately using PBS as elution buffer. The first six fractions of 0.5 mL each were discarded and 7–12 consecutive fractions were collected. Then, fractions 7-9 and fractions 10-12 were combined and concentrated to 0.1 mL using an Amicon Ultra-4 centrifugal filter with 10K MWCO. The EV size was determined by Dynamic Light Scattering and EV concentration was determined using qNano instrument. Fractions were stored at -80°C for subsequent Scanning Electron Microscope (SEM) and DLL3 mRNA and mProtein detection.

**Tunable resistive pulse sensing (TRPS)**

The qNano Gold (Izon Sciences, Boston, MA) was employed to quantify the size and concentration of EVs via NP200 (50 – 330 nm) nanopore membranes. A pressure of 10 mbar and a voltage of 0.48 were applied. Polystyrene nanoparticles (CPC100) were used to calibrate the samples.

**Preparation of Tethered Lipid Nanoparticles (TLN) with or without MBs**

Biotin linked cationic lipid nanoparticles were prepared as described previously with minor modifications. The lipid composition used for TLN was DOTAP/DOPC/cholesterol/ biotin-PEG2000-DSPE at molar ratio of 50/18/30/2. Lipid stock solution was prepared at 10 mg lipid/ml in ethanol. Briefly, 1 μl DDL3 MB (100 μM) and 6 μl of scramble oligonucleotide (300 μM, 21-oligonucleotide) was first mixed with 23 μL PBS and then mixed with 20 μL lipid stock solution. Biotin linked lipid nanoparticles without MB was prepared by mixing 6 μL of scramble oligonucleotide with 24 μL PBS, followed by mixing with 20 μL lipid stock solution. After 5 min of sonication, the mixture was then injected into 450 μL PBS and further sonicated for 1 min at room temperature. The nanoparticle solution were then dialyzed against PBS buffer at room temperature for 2 h using a MWCO 20,000 Dalton dialysis device to remove residual free MB and scramble oligonucleotide.

**Detection of DLL3 mRNA by TLN assay**

The overall concept of the tethered lipoplex nanoparticle (TLN) assay is to capture individual EVs using cationic lipoplex nanoparticles containing MB. Briefly, TLN biochip were washed with PBS and treated with 0.1 mg/ml NeutrAvidin solution (20 μl/well) for 30 min under the shaking condition (Titer Plate Shaker, Speed =2.5). After wash with PBS, 20 μl of lipid nanoparticle with MB were added to each well of the biochip. Then, the biochip was incubated 20 min at room temperature under shaking. The biochip were washed with PBS and then loaded with 20 μl of EV. After 2h incubation at 37°C, the biochip were washed and filled with PBS. Total Internal Reflective Fluorescence (TIRF) microscopy (Nikon Eclipse Ti Inverted Microscope System) was used to detect the DLL3 fluorescence signals from the samples.

**Detection of DLL3 mProtein by TLN assay**

TLN biochip were washed with PBS and treated with 0.1 mg/mL NeutrAvidin solution (20 μL/well) for 30 min under the shaking condition (Titer Plate Shaker, Speed = 2.5). After wash with PBS, 20 μL of cationic lipid nanoparticle without MB were added to each well of the biochip. Then, the biochip was incubated 20 min at room temperature under shaking. The biochip were washed with PBS and then loaded with 20 μL of EV. After incubate for 2 h at 37°C, the biochip was blocked with 4% (w/v) BSA solution for 1 h at room temperature. Then human DLL3 PE-conjugated antibody (1:500 dilutions) in 1% (w/v) BSA PBS solution was added to each well on the biochip and incubated for 1 h at RT. The biochip was washed with PBS and the fluorescence signals were determined using TIRF microscopy.

**Supplementary Figures**


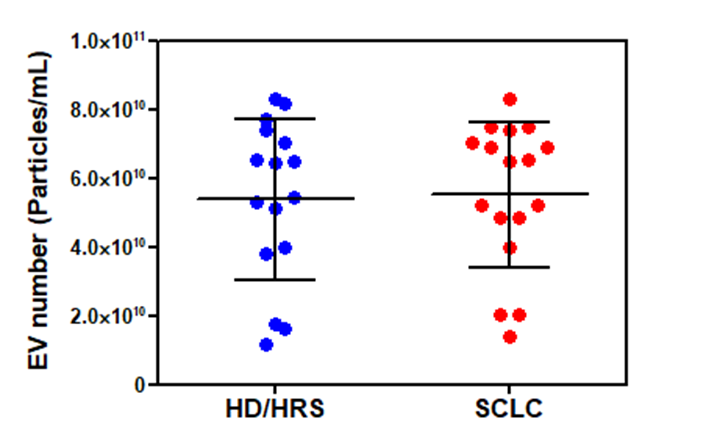


**Figure S1.** **EV concentration measured by tunable resistive pulse sensing**. EVs were isolated using TEI-PK method from Healthy donors (HD), high-risk smokers (HRS), and patients with SCLC.


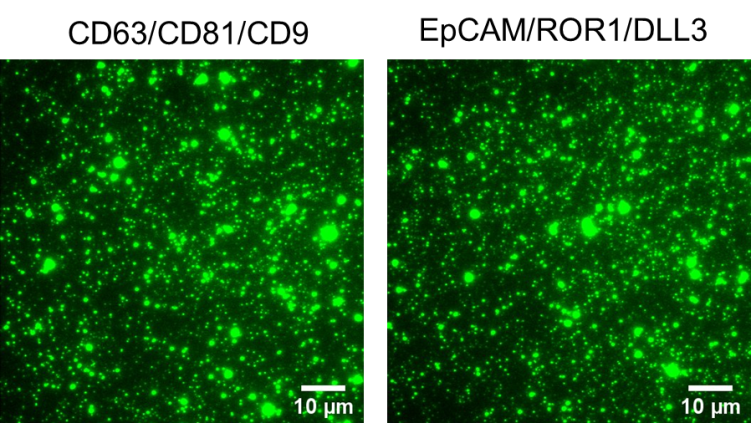


**Figure S2.** **Images of the ILN biochip surface with Saturated EVs.** EVs were labeled in green with PKH67, captured using a mixture of CD63/CD81/CD9 or EpCAM/ROR1/DLL3, and imaged with a TIRF microscope.


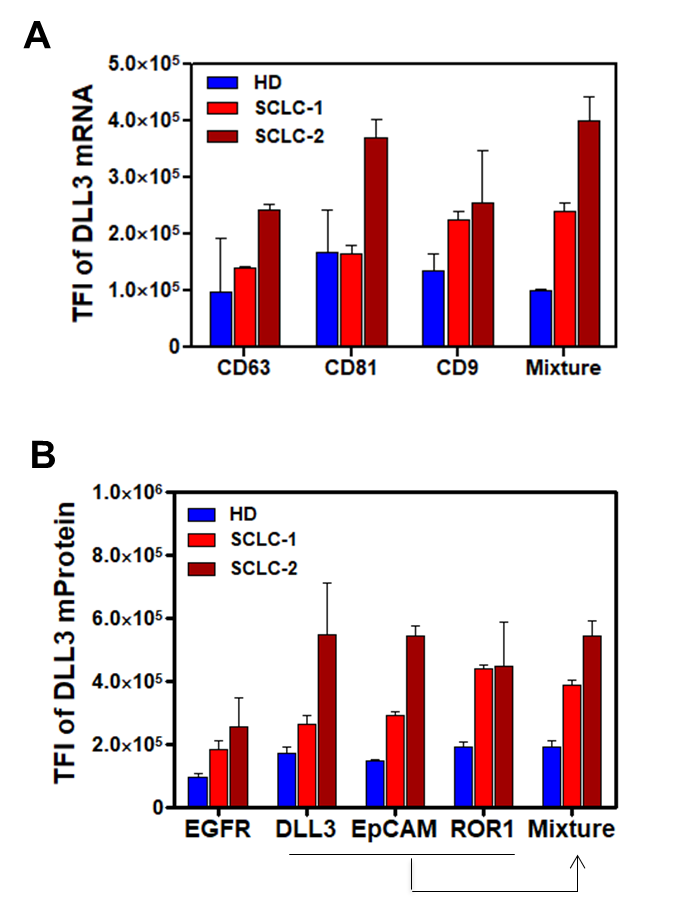


**Figure S3.** DLL3 **(A)** mRNA and **(B)** mProtein expression levels in EVs sorted by individual and mixed capture antibodies from a healthy donor (HD) and two SCLC patient (SCLC-1, SCLC-2) serum samples.


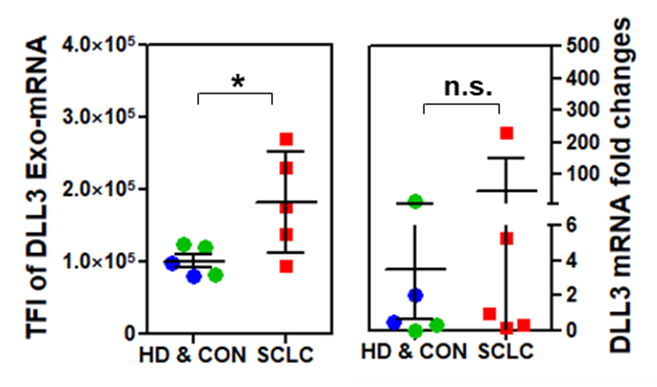


**Figure S4. Comparison of ILN assay and qRT-PCR for EV DLL3 mRNA expression in SCLC and non-patient serum samples.** ILN (left) vs. qRT-PCR (right) for EV DLL3 mRNA. Blue dots are healthy donors (HD), red dots are patients (SCLC), and green dots are control of high-risk smokers (CON). All data are presented as means ± SD (*n* = 2 wells).


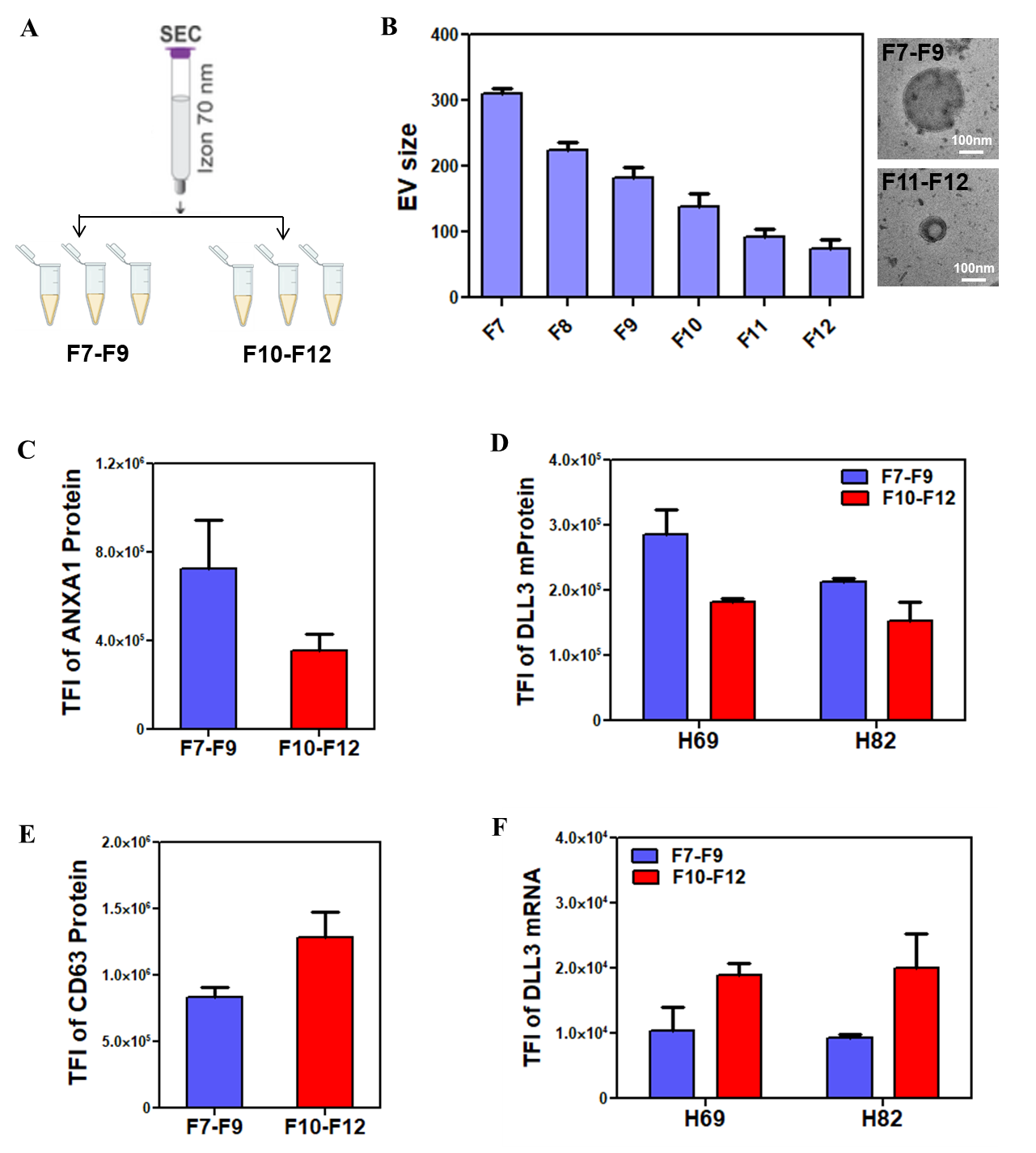


**Figure S5. EV markers, DLL3 mRNA, and mProtein levels in large and small EVs isolated using size exclusion chromatography (SEC) with a qEV column. (A)** Schematic of EV isolation by SEC. **(B)** EV sizes in different qEV fractions measured by dynamic light scattering goniometry (DLS). Insets are representative EV images in F7-F9 (large) and F10-12 (small). **(C)** ANXA1 levels in large (F7-F9) and small (F10-F12) EVs from H82 cells. **(D)** DLL3 mProtein levels in large (F7-F9) and small (F10-F12) EVs from H69 and H82 cells. **(E)** CD63 protein levels in large (F7-F9) and small (F10-F12) EVs from H82 cells. **(F)** DLL3 mRNA levels in large (F7-F9) and small (F10-F12) EVs from H69 and H82 cells. All data are presented as means ± SD (*n* = 2 wells).


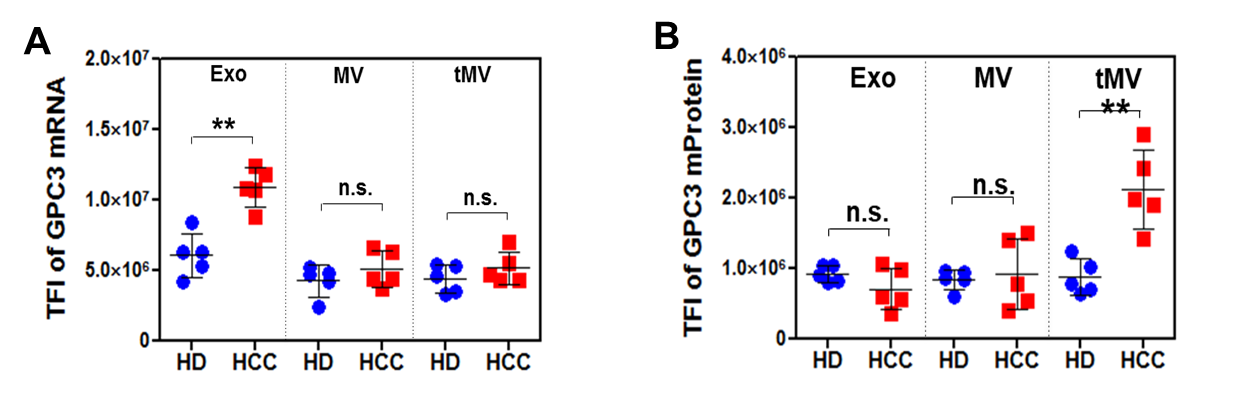


Figure S6. GPC3 mRNA and mProtein expression levels in different EV subpopulations from hepatocellular carcinoma (HCC) (*n* = 5) and healthy donor (HD) (*n* = 5) serum samples.


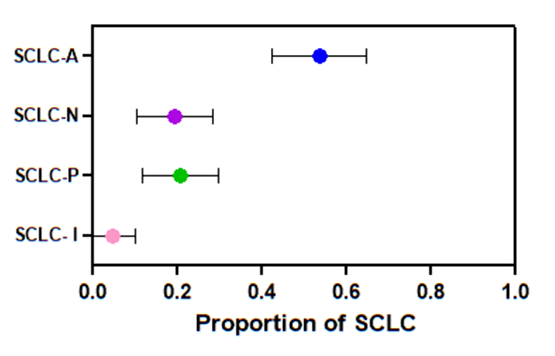


**Figure S7.** Estimates of relative frequencies of the four subtypes based on ILN assay with 95% CIs (*n* = 76).


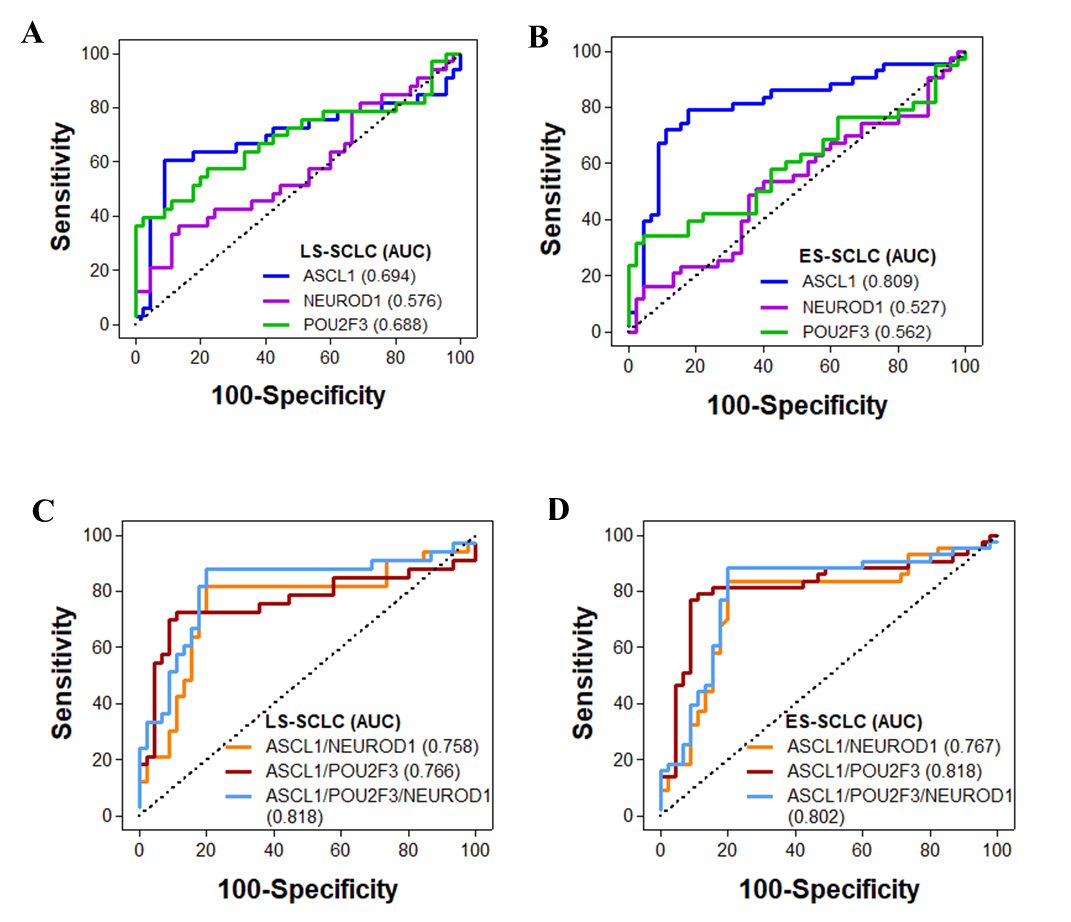


**Figure S8.** AUC/ROC curves of subtype transcription factors in SCLC patients with limited-stage (**A,C**) and extensive-stage (**B,D**) as single biomarker (**A,B**) or combined biomarker (**C,D**).
